# Human oral cells’ response to different endodontic restorative materials: an *in vitro* study

**DOI:** 10.1186/s13005-014-0055-4

**Published:** 2014-12-23

**Authors:** Susanne Jung, Jana Mielert, Johannes Kleinheinz, Till Dammaschke

**Affiliations:** Department of Maxillofacial Surgery, Albert-Schweitzer-Campus 1, Building W30, 48149 Münster, Germany

**Keywords:** Amalgam, Biodentine, Composite resin, Mineral Trioxide Aggregate, Osteoblasts, Periodontal ligament cells

## Abstract

**Introduction:**

The aim of this study was to compare the biological interaction of human osteoblasts and cells of the human periodontal ligament (PDL) with different endodontic restorative material as Mineral Trioxide Aggregate (MTA), Biodentine, amalgam and composite over a time period of 20 days.

**Materials and methods:**

Human PDL cells and osteoblasts were harvested, cultured and according to standardized protocols. The cell populations were characterized with the corresponding surface markers following standardized procedures. The specimens were produced with special regard to constant dimensions and volume in the different groups. Cell attachment and proliferation were evaluated morphologically after Richardson staining and cell count was performed after 1d, 8d, 13d and 20d. All experiments were performed in triplets. The results were statistically analyzed using the ANOVA- and Tukey-test (p < 0.05).

**Results:**

Morphological analysis proved good proliferation and cell attachment in both cements. A remarkable result was the organized spreading and parallel alignment of the PDL cells in contact with MTA and especially Biodentine (cells maturing in a second cell layer crossway to the first one). From 8d onward Biodentine showed the highest quantity of PDL cells (p < 0.05). Biodentine and MTA resulted in a significantly higher cell density in osteoblast and PDL cell culture. The other groups showed a lower PDL cell density from 8d and a lower osteoblast cell density from 13d when compared to control and cement samples (p < 0.05).

**Conclusions:**

MTA and Biodentine showed a good biocompatibility in contact with the human osteoblasts and cells of the periodontal ligament. Regarding cell survival and proliferation particularly of PDL cells Biodentine showed good results and can be considered as a well-tolerated bioactive endodontic material.

**Electronic supplementary material:**

The online version of this article (doi:10.1186/s13005-014-0055-4) contains supplementary material, which is available to authorized users.

## Introduction

During endodontic therapy a perforation of the root canal system may occur or a root-end surgery may be necessary. Both procedures resulting in communication of the pulp chamber or the root canal system with the periodontium. For the best prognosis, these contact areas must be restored and sealed. Hence, aim of such filling is to obturate the root-end or perforation cavity and to prevent micro leakage. A suitable filling material should:

- be biocompatible;
- ensure a long-term three-dimensional sealing of all margins, preferably by a molecular bonding to the dentinal walls;
- be bacteriostatic, or not encourage bacterial growth;
- be stable;
- be insoluble;
- be non-absorbable;
- be not moisture-sensitive;
- be easy to prepare and place;
- be radiopaque and
- be bioactive and induce regeneration of the periodontal ligament and bone [1-4].

Nevertheless, for a successful endodontic therapy a high quality apical root canal filling or perforation repair is essential. In the past, numerous different materials like amalgam, reinforced zinc oxide eugenol cements (IRM, Super-EBA), glass ionomer cement, and composite resin were used to fill endodontic perforations or as root-end filling material [2,4-10]. Unfortunately, an ideal root-end filling material has yet not been found [2,4].

In the recent years Mineral Trioxide Aggregate (MTA), a refined Portland cement [11,12], was extensively tested for this propose and was found to provides distinctly less cytotoxic effects and better results concerning material properties, biocompatibility, microleakage protection, bioactivity and thus, clinical success than traditional materials recommended for root-end fillings or perforation repair. Due to its good biocompatibility, mechanical stability and regenerative impact on hard tissue and periodontium, ProRoot MTA is denoted as a reference material for root perforation repair and root-end obturation [13-15].

Even though ProRoot MTA (Dentsply/Tulsa, Tulsa, OK, USA) appears to be the preferred material in the above mentioned indications with many positive features, the cement does have several drawbacks: the handling can be difficult, the setting time is long, the use in the visible crown area may lead to tooth discoloration, the compressive and flexural strength is lower than dentine and it is quite expensive [4,16-18].

Recently, a new bioactive calcium silicate cement, Biodentine (Septodont, St. Maur-des-Fossés, France), was launched on the dental market. Biodentine consists of a powder in a capsule and liquid in a pipette. According to Camilleri et al. the powder consists mainly of SiO_2_ (16.90%), CaO (62.9%), ZrO_2_ (5.47%), and the liquid is composed of Na (15.8%), Mg (5.0%), Cl (34.7%), Ca (23.6%), and H_2_O (20.9%) [19].

Compared to ProRoot MTA until now comparatively little information about Biodentine is available. Used as root-end filling Biodentine showed clinically a good bony regeneration after apicoectomy [20]. When comparing its material characteristics to established tricalcium silicate cements Biodentine stands out by its greater compressive strength, most likely caused by the low water/cement ration of the mixture. The material is described less porous and denser than MTA; the alkaline pH of Biodentine is comparable to other cements. Investigations of the microleakage revealed that tracer diffusion between dental material and dentin walls was significantly reduced in Biodentine samples compared to glass ionomer cement and MTA. The colour stability of Biodentine allows its appliance in aesthetically susceptible areas [21].

Nevertheless, the selection of a repair material is critical because biocompatibility and sealing ability are reported to have an effect on the prognosis of perforation closure or apicoectomy [22]. The biocompatibility of endodontic materials is essential because during application the materials/cements might get direct contact to the surrounding bone or the periodontium for a prolonged period of time. Periodontal ligament (PDL) fibroblasts with specialized functions are responsible for the formation and maintenance of PDL fibre attachments as well as repair, remodelling, and regeneration of the adjacent alveolar bone and cementum [23]. PDL cells are responsible for normal maintenance and regeneration of the PDL [24]. In addition to PDL fibroblasts, cells from the surrounding alveolar bone are likely to play an important role in the repair and regeneration of periradicular tissue [25]. PDL cells are usually formed around the root-end or perforation filling materials [26]. Osteoblasts and PDL cells are the principal cells responsible for osseous excisional wound healing after periradicular surgery [27].

Thus, the aim of this investigation was to analyze the biological interaction of human osteoblasts, and cells of the PDL with different endodontic restorative materials: Mineral Trioxide Aggregate cement (ProRoot MTA), Biodentine (as another calcium silicate cement), amalgam, and composite resin up to 20 days *in vitro*. Amalgam and composite were included in the study design to analyze their biological effects on cells in direct proximity to further clarify their influence on cell proliferation in an *in vitro* setting.

The null-hypotheses of this study were that Biodentine will show biocompatible reaction to PDL cells and osteoblasts comparable to ProRoot MTA, whereas amalgam and composite resin will have a negative impact.

## Materials and methods

### Sample preparation

The following materials were included in this study: ProRoot MTA (Dentsply/Tulsa, Tulsa, OK, USA), Biodentine as other calcium silicate cement (Septodont, Saint-Maur-des-Fossés, France), a light-curing composite resin (Estelite Σ Quick; Tokuyama Dental, Tokyo, Japan) and an amalgam (Oralloy Magicap S; Coltène/Whaledent, Altstätten, Switzerland).

From all materials samples were produced with a defined diameter of 5 mm and a height of 2 mm. All materials were handled strictly according to manufacturer recommendations. The samples were prepared with consideration of their specific curing processes: while Biodentine sets for 12 minutes, MTA sets for four hours and amalgam for 24 hours. The composite samples were light cured in layers (incremental technique).

The human cells were harvested and cultured according to a standardized protocol. All cell samples were taken after the patients’ informed consent. The Ethics committee of the Westphalian Wilhelms-University, Münster, Germany approved the use of human cells (Reg. No. 1IXKlei1). The handling of all human samples followed strictly the “Declaration of Helsinki”.

Primary osteoblasts were harvested from bone chips collected during modelling mandibular osteotomies or the surgical removal of lower wisdom teeth. The bone particles were cultured in MM0 medium (High Growth Enhancement Medium; MP Biomedicals, Eschwege Germany) with fetal bovine serum, Penicillin (10.000U/ml), Streptomycin (10.000 μg/ml) and Amphotericin B 250 μg/ml (Biochrom, Berlin, Germany). After 10 days dexamethasone (Merck, Darmstadt, Germany; 0.02% in phosphate buffered saline (PBS Dulbecco, Biochrom, Berlin, Germany) was added to the medium.

The outgrowing cells were characterized immunohistochemically by positive expression of osteocalcin, osteonectin and collagen I. The second passage was used for the experiments.

The human periodontal ligaments cells were harvested from the periodontal membrane of impacted, surgically removed wisdom teeth, which therefore did not have any contact with the oral cavity at any time. The PDL cells were cultured in Dulbeco’s Modified Eagle Medium 1X (Lot 1012067, 4,5 g/l Glucose, L-Glutamine, Pyruvate; gibco by life technologies, Darmstadt, Germany) with fetal bovine serum, Penicillin (10.000U/ml), Streptomycin (10.000 μg/ml) and Amphotericin B 250 μg/ml (Biochrom, Berlin, Germany).

The material samples were placed in 6-well-dishes (TPP, Trasadingen, Switzerland) and brought in direct contact to the harvested cell. The cells were plated at a density of 5,000 cells/cm^2^ and cultured in their respective cell culture medium (PDL cells in Dulbeco’s Modified Eagle Medium 1X and osteoblasts in MM0, High Growth Enhancement Medium with dexamethasone and in the presence of the samples. Cells at passage P2 were used for the study. All experiments were performed in triplets. The *in-vitro* investigations were performed after defined intervals of one, eight, thirteen and twenty days. To control the growth and proliferation of the cells also cultures without contact to any test material were assessed (control group).

### Morphological analysis

For histological evaluation the cell cultures were fixed in methyl alcohol (Merck, Darmstadt, Germany) at 21°C room temperature, air dried and a Richardson staining was performed. The Richardson solution ready for use consisted of two filtered stock solutions. Stock solution I is 1% methylen blue in 1% sodium borate. The stock solution II is a solution of 1% azure in distilled water (Merck, Darmstadt, Germany). The working solution is prepared on day of use by mixing the stocks 1:1 and stored in a syringe with a 22 μm filter. For the dyeing of the cell cultures the working solution was warmed to 60°С, two drops of the solution were given to every deepening and the slides were stained for one minute. They were subsequently washed in bidistilled water, air dried and mounted. With the help of light microscopic images of the cell cultures in the Richardson’s stain the morphology and proliferation of the cells were evaluated.

### Cell count

After siphoning the culture medium, the samples were washed twice with PBS, covered with a thin film of 1 ml trypsin (0.05%)/EDTA (0.02%) solution (Biochrom, Berlin, Germany) and incubated in the incubator for three minutes. The reaction was stopped by admixing 2 ml of the fresh corresponding medium and the cells were resuspended by thoroughly pipetting up and down several times.

100 μl of the cell suspension of each deepening was pipetted into a Coulter-potty (Beckman Coulter GmbH, Krefeld, Germany) filled with 10 ml isotone electrolyte solution (CasyTon; Schärfe System, Reutlingen, Germany). A cell count was performed with the Casy 1 Cell Counter & Analyzer system (Schärfe System, Reutlingen, Germany).

The proliferation of the osteoblasts and the PDL cells was considered and evaluated referring to the determined absolute cell counts in the presence of the four materials as well as in their absence. The cell colonies were assessed based on the morphological analysis of the cell size, the morphology of the nucleus and the proliferation. All results were statistically analyzed using the ANOVA and Tukey test (p < 0.05).

## Results

### Morphological analysis

#### Osteoblasts

**Figure 1:**
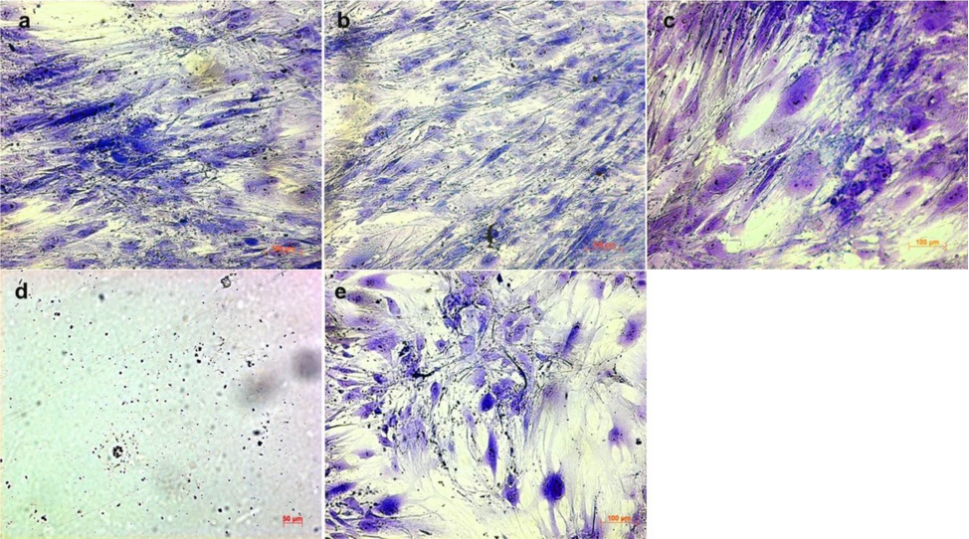
**Human osteoblasts after contact with different endodontic restorative materials for 20 d.** (Richardson staining, ×100; **a**, ProRoot MTA; **b**, Biodentine; **c**, amalgam; **d**, composite resin; **e**, control).

When analyzing the morphology under human osteoblasts’ interaction with ProRoot MTA, Biodentine and amalgam as well as with the control group, the histological imaging was homogenous. An increasing density of the cells was observed for all cell cultures in the presence of these materials. At the same time, an increasing number of polygonal cells were noticed (Figure 1a-c, e). The cell reactions to the presence of polymerized composite resin were in strong contrast to these results: the histological images illustrated structural decomposition up to numerous cell losses (Figure 1d).

#### PDL cells

**Figure 2:**
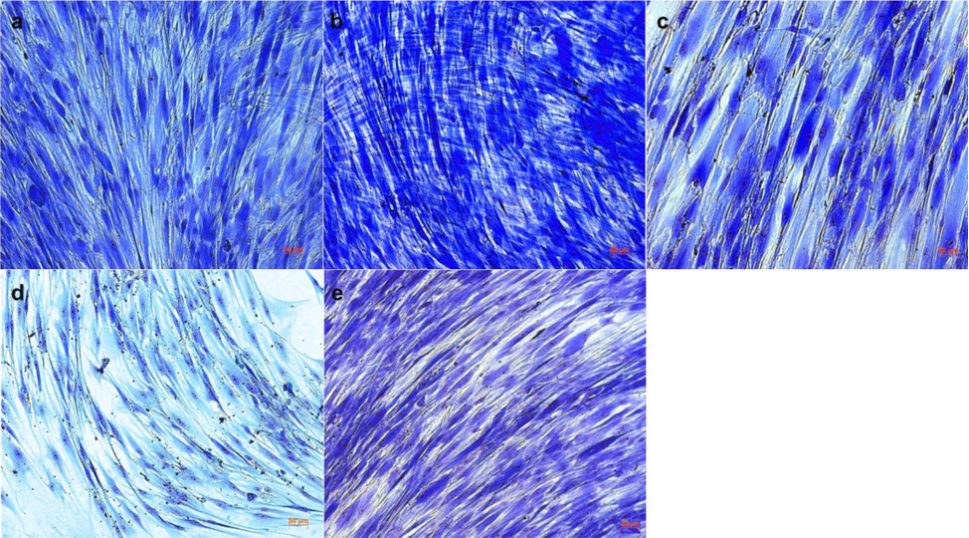
**Human PDL cells after contact with different endodontic restorative materials for 20 d. for 20 d.** (Richardson staining, ×100; **a**, ProRoot MTA; **b**, Biodentine; **c**, amalgam; **d**, composite resin; **e**, control).

During the first days of cell culture, the PDL cells appeared as long, slender cells without an ordered arrangement. The cells were similar to fibroblasts. In addition to that, polygonal cells could occasionally be identified. In the further course of the cultivation a remarkably strong growth of the cultures took place so that after twenty days a confluent monolayer could be recognized in all cultures. An exceptional result was the organized spreading and the strictly parallel alignment of the cells (Figure 2a-e). The presence of Biodentine had impressive stimulatory effects. Only in this group beyond the forming of a confluent cell layer, the PDL cells matured in a second cell layer crossway to the first one (Figure 2b). In contrast the culture under the influence of the polymerized composite resin showed reduced cell density and growth. An increase of cells had occurred and they ordered themselves in a parallel arrangement, but no confluent monolayer was formed after 20 d (Figure 2d).

### Cell count

#### Osteoblasts

**Figure 3:**
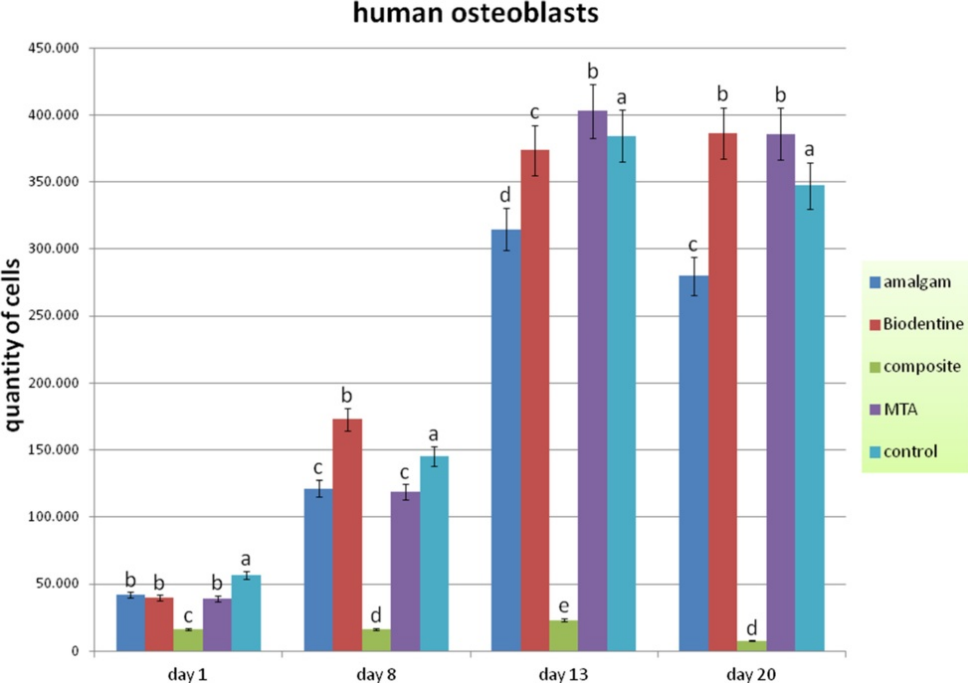
**Quantity of human osteoblasts after contact to different endodontic restorative materials up to 20 d.** (Different characters above bars indicate a statistical significant difference between the number of cells within one observation day [p < 0.05]).

One day after the application of the human osteoblasts onto the material samples the cell quantity was significantly decreased in all groups compared to the untreated control group (p < 0.05). In the following days it became obvious that the light cured composite resin samples had a negative impact on the osteoblasts. The number of cells dropped considerably. In all other groups the amount of cell increased within one week. After that time the Biodentine group showed significantly more osteoblasts than all other group (p < 0.05), whereas after 13 d significantly more cells could be detected in the ProRoot MTA group (p < 0.05). After 20 d a slight reduction in the cell amount was visible in all groups. However, Biodentine and ProRoot MTA showed significantly more cells than all other groups. Already after 8 d Biodentine showed a significant higher quantity of cells compared to the control group, whereas in the amalgam group the amount of cells was significant lower compared to the control group at all days (p < 0.05) (Figure 3).

#### PDL cells

**Figure 4:**
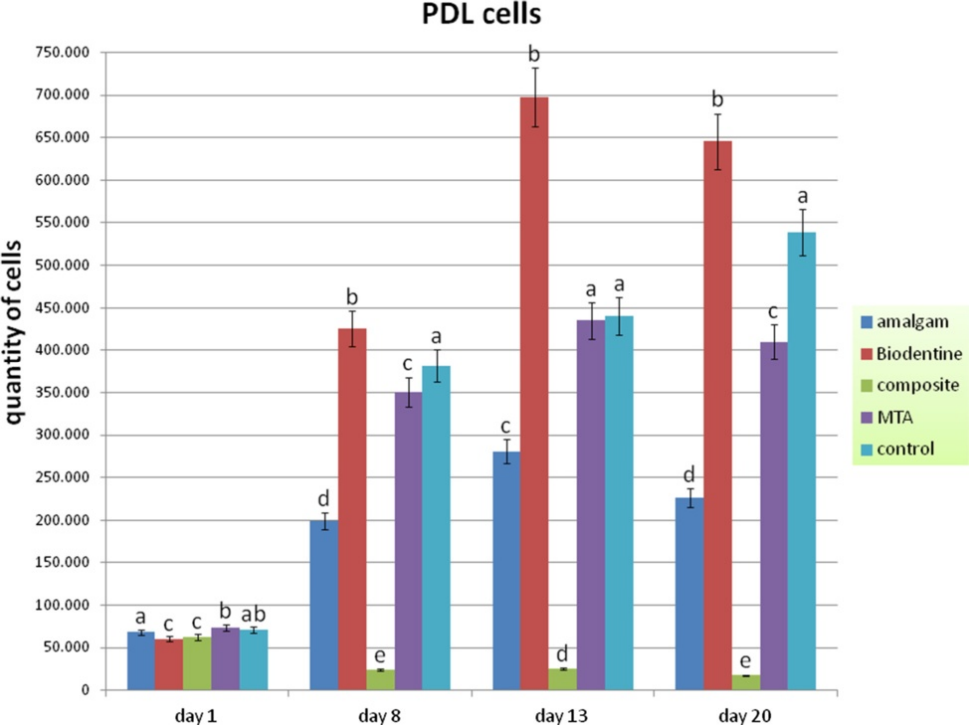
**Quantity of human PDL cells after contact to different endodontic restorative materials up to 20 d.** (Different characters above bars indicate a statistical significant difference between the number of cells within one observation day [p < 0.05]).

The application of the material samples to PDL cells had not that impact on the cell quantity than it has on the osteoblasts after one day. In the amalgam and in the ProRoot MTA group the cell quantity was statistically not significantly different from the control group (p > 0.05). Comparable to the osteoblast culture the composite resin showed a strong negative effect on PDL cells (p < 0.05). Thus, the amount of cells dropped considerably. Also comparable to the osteoblasts the amount of PDL cells clearly increased in all other groups after one day. Nevertheless, after 8 d and 20 d in contact with amalgam and ProRoot MTA the amount of cells was significantly lower compared to the control group (p < 0.05) whereas from 8 d onward Biodentine showed a significantly higher quantity of PDL cells compared to all other groups (p < 0.05). After 13 d a difference in the cell number could not be detected between the controls and ProRoot MTA (p > 0.05) (Figure 4).

## Discussion

The most ideal healing outcome after filling the resected root canal or the perforation would be reformation of a normal attachment apparatus with healthy bone, periodontal ligament, and cementum [27]. Hence, the ultimate goal of treatment of root perforations or root-end surgery is to maintain or re-establish the damaged attachment apparatus [28]. Damage of the PDL will have adverse effects on healing following endodontic surgery, bony regeneration and may lead to an unfavourable outcome of treatment. Hence, to evaluate the biocompatibility and bioactivity of a new calcium silicate cement (Biodentine) in comparison to ProRoot MTA human osteoblasts and PDL cells were chosen for this *ex vivo* study. The results from studies of these cells are favourable to those from other cell lines (e.g. osteosarcoma cells) or cells of animal origin because their reaction concerning cell attachment and mineralization may be different to human osteoblasts or PDL cells [24,25].

In the resent years MTA has been extensively examined in dental science and numerous cytotoxicity and cell attachment investigations with various cell cultures showed better results with MTA in comparison to very many other dental materials [14]. Nowadays, the use of MTA can be assumed as gold standard for the closure of perforations defects or as root-end filling against which other materials need to be tested. The very good biocompatibility and bioactivity of MTA on PDL cells and osteoblasts are confirmed in the present study and are in fully accordance with the present knowledge about MTA [13-15].

Whereas MTA is very well investigated also on human cell lines [14] to the best of our knowledge until today no data are published concerning the effect of Biodentine to human osteoblasts or PDL cells. Only Zhou et al. compared Biodentine and MTA in direct contact on human gingival fibroblasts. Both cements showed no significant differences in cell viabilities. The cells attached to and spread over both material surfaces [29].

When comparing Biodentine and its characteristic properties to other well established dental materials as Super EBA or glass ionomer cement one has no recourse to much experimental data in current literature. In an investigation of 2012 Al-Hiyasat et al. observed the quality of cellular attachment to various root-end filling materials and concluded that the best cellular attachment of fibroblasts can be observed on the surface of MTA, whereas Super EBA surfaces did not attract cell adherence most likely due to the leaking of eugenol into the dentinal tubules. Unwashed glass ionomer cement surfaces did not induce cell attachment either [30]; these findings support our data concerning the biological effect of MTA. In a direct comparison of Biodentine and glass ionomer cement as dentine replacement material by Camilleri in 2013, glass ionomer cements showed more physical and chemical stability and led to significantly less microleakage when applied under composite restorations in a sandwich technique. The indication and application for each material have to be well considered [31].

The results of the present study emphasize that Biodentine - beside MTA - can be called a bioactive cement by up-regulating osteoblasts and PDL cells activity. Biodentine can be considered as a material that may induce periodontal regeneration and/or repair. Biodentine has favourable properties regarding biologic response of the cells within the periodontium which were evaluated in this study. Only in contact to Biodentine the PDL cells matured in a second cell layer crossway to the first one. And from 8 d onward Biodentine showed the statistically significant highest quantity of PDL cells.

Biodentine is mainly composed out of tri- and dicalcium silicate. Recent research in medicine clearly showed that the addition of tricalcium silicate to calcium phosphate bone cements improves the bioactivity of those materials on osteoblast or osteoblast like cells [32,33]. This may be related to the release of silicon (Si) from calcium silicate cements. It is well known that Si has a positive impact on bone metabolism and enhances the rate of new bone growth when released from bioactive materials *in vivo* [34-36]. These findings suggest that the release of Si from calcium silicate cements may confer additional *in vivo* bioactivity of these materials.

Furthermore, Biodentine and MTA are able to develop a hydroxyl apatite-like surface in the presence of body liquids containing calcium or phosphate [37]. This surface is biocompatible and displays good conditions for cell attachment and proliferation [37,38]. Biodentine showed significantly higher levels of calcium and silicon ion release than MTA [38-40]. Hence, it may be speculated in how far the higher release of Ca and Si from Biodentine may explain the present findings.

For many years, amalgam was accepted as the material of choice in endodontic surgery but it came into question when concerns about its toxicity arose [1,41,42]. Hence, amalgam is far from being an ideal restorative material in endodontics as a consequence of its potential disadvantages: beside the potential biological toxicity of its constituents, initial leakage, moisture sensitivity, and need for an undercut in the cavity preparation are mentioned [1,41,42]. Thus, *in vivo* amalgam used as material to repair furcation perforations leads to severe inflammatory response in the furcal bone [43]. In contact to PDL cells amalgam showed a mild [44] to severe [45] cytotoxicity showing early manifestation of cell injury [25]. Thus, Zhu et al. found a significantly lower total cell number of osteoblasts and PDL cells in direct contact to amalgam compared to the untreated control group after 3 d and 7 d [46].

Compared to MTA in direct contact to human PDL cells amalgam was significantly more cytotoxic within 1 d and the PDL cell density was lower after 4 d [47,48]. After 72 h direct contact to amalgam the number of osteoblast-like cells was significantly lower than after contact to MTA. Whereas the number of the cells in the MTA group was significantly lower than that in the untreated control group [49].

The result of this study showed that the negative impact amalgam on human PDL cells and osteoblasts was not as distinct as hypothesized. Nevertheless, because the quantity of PDL cells and osteoblasts were significantly lower than in the control group at all days, amalgam showed no bioactivity and thus cannot be recommended as filling material in endodontic surgery.

Only limited reports about the influence of composite resins on cells associated with bone formation and periodontal repair are available [50]. Hence, *ex vivo* studies like the present are of some importance. It may be concluded from the results of the present study that the direct contact of PDL cells and osetoblasts to composite resin should be avoided because cell proliferation is suppressed.

Comparable to the present study Tai and Chang found that composite resin exhibited the most toxic effects followed by amalgam. Thus, amalgam reacts more favourable to PDL cells than composite resin [28].

In contrast to the present study Zhu et al. showed osteoblasts attached and spread on composite resin as well as on MTA by forming a monolayer [51] and cementum with inserting collagenous fibres from the periodontal ligament have been reported to form on composite resin *in vivo* [52]. Nevertheless, composite resin showed clearly cytotoxic effects on osteoblasts [53]. Thus, a variety of studies investigating cytotoxic effects of composite resin monomers on mammalian cells have demonstrated that unreacted resin monomers from rein-based materials can cause adverse biological effects on oral tissue. Hence, dental resin monomers showed genetic and cellular toxicology. Unpolymerized monomers released from cured composite resin may hamper the healing process of surrounding tissue. Thus, the usage of such materials in direct contact with bone, periapical or periodontal tissue is questionable [50,53-55].

In the present investigation the leachable components in the culture medium were not assessed. Nevertheless, cytotoxic monomers are eluted from light cured composite resin in a significant extent [50,56].

The null-hypothesis of this investigation could not be fully confirmed: The effect of Biodentine on osteoblast was comparable to ProRoot MTA whereas quantity of PDL cells after contact to Biodentine was significantly higher compared to ProRoot MTA from day 8 onward. The quantity of human osteoblasts and PDL cells after contact to amalgam was significantly lower compared to the control but significantly higher compared to the composite resin. Thus, both materials had a negative impact to the tested cells, whereas the results in the composite group were significantly worse. Only in the composite resin group a remarkable structural changes up to numerous cell losses, reduced cell density and growth could be observed but no confluent monolayer.

## Conclusion

ProRoot MTA and Biodentine showed no cytotoxicity and a good biocompatibility in direct contact with osteoblasts and PDL cells. Regarding cell survival and proliferation particularly of PDL cells Biodentine showed good results and can be considered as a well-tolerated endodontic material with stimulatory bioactive properties. Amalgam and especially composite resin might not provide an appropriate environment for osteoblasts and PDL cells.
